# The development and initial validation of self‐report measures of ICD‐11 depressive episode and generalized anxiety disorder: The International Depression Questionnaire (IDQ) and the International Anxiety Questionnaire (IAQ)

**DOI:** 10.1002/jclp.23446

**Published:** 2022-10-10

**Authors:** Mark Shevlin, Philip Hyland, Sarah Butter, Orla McBride, Todd K. Hartman, Thanos Karatzias, Richard P. Bentall

**Affiliations:** ^1^ School of Psychology Ulster University Coleraine Northern Ireland; ^2^ Department of Psychology Maynooth University Maynooth Ireland; ^3^ Department of Social Statistics University of Manchester Manchester England; ^4^ School of Health & Social Care Napier University Edinburgh Scotland; ^5^ Department of Psychology University of Sheffield Sheffield England

**Keywords:** anxiety, depression

## Abstract

**Background:**

The new International Classification of Diseases came into effect in 2022 (ICD‐11; World Health Organization, 2022) and included updated descriptions and diagnostic rules for “Depressive Episode” and “Generalized Anxiety Disorder.” No self‐report measures align with these disorders so this study reports the development and initial validation of the “International Depression Questionnaire” (IDQ) and “International Anxiety Questionnaire” (IAQ).

**Methods:**

Items were developed that aligned to the ICD‐11 descriptions and their performance was assessed using data from a community sample (*N* = 2058) that was representative of the United Kingdom adult population.

**Results:**

Item response theory models indicated that the two scales were unidimensional, and the items performed well in terms of difficulty and discrimination. Estimates of internal reliability were high. Based on ICD‐11 derived diagnostic algorithms, 7.4% met requirements for ICD‐11 Depressive Episode and 7.1% for Generalized Anxiety Disorder.

**Conclusions:**

The IDQ and the IAQ are short, easy to use, self‐report measures aligned to the new and updated ICD‐11 diagnostic descriptions. This study provides initial evidence that the scales produce scores that are reliable and valid.

## 1 INTRODUCTION

There is a plethora of self‐report measures of depression and anxiety. Santor et al. (2006) reported that since 1918 “…more than 280 measures of depressive severity have been developed and published” (p. 135), and it is likely that there is a similarly large number of measures of anxiety. However, these measures of each disorder are unlikely to be interchangeable as Fried (2017) reported low levels of content overlap in common measures of depression, and similar findings have been reported for measures of anxiety (Wall & Lee, 2021). The implication is that different scales intended to capture the same disorder are likely measuring different constructs, or at a minimum, different aspects of the same constructs. There is also evidence of inconsistency in the identification of probable diagnostic cases between the measures (Cameron et al., 2008; Peters et al., 2021). Fried (2017) noted that this can result in bias, misclassification, problems with replication, idiosyncratic findings, and lack of comparability across studies.

A relatively simple approach to improve the assessment of anxiety and depression is to ensure that measures correspond to the symptoms and diagnostic requirements described in the main diagnostic manuals, namely the International Classification of Diseases (ICD‐11: World Health Organization [WHO], 2022) and the Diagnostic and Statistical Manual of Mental Disorders (DSM‐5: American Psychiatric Association [APA], 2022). The idea of closely aligning the content of a measure to diagnostic requirements was the basis for the widely used Patient Health Questionnaire‐9 (PHQ‐9: Kroenke et al., 2001) and Generalized Anxiety Disorder‐7 (GAD‐7: Spitzer et al., 2006), which were developed to measure symptoms of DSM‐IV (APA, 1994) Major Depressive Disorder and Generalized Anxiety Disorder, respectively. Given their alignment with diagnostic descriptions, their terse nature, and being freely available, these scales have become the de facto research measures of depression and anxiety. They are also commonly used in clinical settings. For example, in the United Kingdom (UK) the PHQ‐9 and GAD‐7 are used to identity individuals in need of access to psychological therapy, and to assess symptom change before and after therapy (NCCMH, 2018). These scales have proven to be hugely successful and continue to offer great value today. However, they were developed before the release of the most recent editions of the DSM (i.e., DSM‐5) and ICD (i.e., ICD‐11) and therefore no longer wholly align to current diagnostic requirements (for review of similarities/differences in ICD‐11 and DSM‐5 see Stein et al., 2020; and First et al., 2021).

There are, in fact, currently no measures that reflect the symptom descriptions and diagnostic requirements set out in the ICD‐11 for Depressive Episode (diagnostic code: 6A70) and Generalized Anxiety Disorder (diagnostic code: 6B00). This is problematic because the ICD‐11 is the diagnostic manual used by all WHO member states (including the United States) as of January 1, 2022 to collect mortality and morbidity statistics, and anxiety and depression are the most common mental health disorders, globally, as well as the two most disabling mental health disorders (GBD 2019 Mental Disorders Collaborators, 2022). Thus, for the accurate tracking of these disorders in line with the global standard for coding diagnostic health information, it is imperative that measures be available to researchers and clinicians that capture anxiety and depression symptoms, severity, and ‘caseness’, as per the ICD‐11. It is also important as Depressive Episode is integral to the diagnosis Recurrent Depressive Disorder (diagnostic code 6A71), as well as Bipolar Type I Disorder (diagnostic code 6A60) and Bipolar Type II Disorder (diagnostic code 6A61).

Consequently, the primary aim of this study was to develop self‐report measures that capture the symptoms and diagnostic requirements of ICD‐11 Depressive Episode and ICD‐11 Generalized Anxiety Disorder. The secondary aims were to provide preliminary evidence about the validity and reliability of these scale scores. To do so, validity was assessed by testing the dimensionality of the measures using item response theory (IRT) models, and we hypothesized that the measures would be unidimensional and provide most information above the mean of the underlying dimensions. Reliability was assessed using McDonald's omega (ω: McDonald, 1999). Additionally, prevalence estimates were produced in line with ICD‐11 diagnostic requirements set forth in the ICD‐11 Clinical Descriptions and Diagnostic Requirements (CDDR), and these were compared to criterion demographic (age and sex), mental health (mental health treatment seeking), and clinical (scores and ‘caseness’ on the PHQ‐9 and GAD‐7) variables.

## 2 METHOD

### 2.1 Participants

This study used data collected as part of Wave 6 of the COVID‐19 Psychological Research Consortium (C19PRC) Study, which was established in March 2020 to assess the long‐term psychological, social, and economic impact of the COVID‐19 pandemic on the UK population. Briefly, at baseline (Wave 1, March 23–28, 2020), 2025 adults were recruited via the survey company Qualtrics using quota sampling methods to ensure that the sample characteristics were representative of the UK adult population with respect to age, sex, and 2019 household income. Data for Wave 6 were collected between August 6 and September 28, 2021, during which time data were collected in two stages: at Phase 1 (August 6–September 28), all participants who had previously taken part in the main strand of the C19PRC Study (at baseline or recruited during subsequent waves) were recontacted; at Phase 2 (8–28 September), new participants were recruited to match specific characteristics of the adults lost to panel attrition. This resulted in a recontacted Phase 1 sample of 1643 (51.8% retention rate) and 415 new participants recruited at Phase 2. The combined final Wave 6 sample (*N* = 2058) closely mirrored the characteristics of the baseline sample and was representative of the UK adult population aged 18 years and older with respect to sex, age, and household income (see https://osf.io/qv47z/) Ethical approval for the study was granted by the University of Sheffield (Ref no. 033759). The Wave 6 data used in the current study is available at: https://osf.io/qv47z/ Sample characteristics are reported in Table 1.

**Table 1:** Demographic characteristics of the sample

	*N*	%
*Gender*		
Male	983	47.8%
Female	1069	51.9%
Transgender	4	0.2%
Prefer not to say/other	2	0.0%
*Age*		
18–24	213	10.3%
25–34	395	19.2%
35–44	380	18.5%
45–54	422	20.5%
55–64	354	17.2%
65+	294	14.3%
*Ethnicity*		
White British/Irish	1805	87.7%
White non‐British/Irish	65	3.2%
Indian	42	2.0%
Pakistani	26	1.3%
Chinese	20	1.0%
Other ethnic group	100	4.70%
*Highest qualification*		
No qualifications	61	3.0%
O‐level/GCSE or similar	412	20.0%
A‐level or similar	400	19.4%
Technical qualification	207	10.1%
Undergraduate degree	558	27.1%
Diploma	73	3.5%
Postgraduate degree	322	15.6%
Other qualifications	25	1.2%
*Employment status*		
Employed full‐time	925	44.9%
Employed part‐time	281	13.7%
Self‐employed full‐time	61	3.0%
Self‐employed part‐time	55	2.7%
Unemployed, looking for work	83	4.0%
Unemployed, family or home	128	6.2%
Unemployed, sick or disability	122	5.9%
Government “furlough” scheme	6	0.3%
Retired	342	16.6%
Full‐time student	55	2.7%

### 2.2 Measures

The items, response format, and diagnostic algorithms of the International Depression Questionnaire (IDQ) and the International Anxiety Questionnaire (IAQ)^1^ were derived directly from the ICD‐11 descriptions of Depressive Episode and Generalized Anxiety Disorder. The questionnaires, and their alignment with ICD‐11 descriptions, are presented in Tables 2a and 2b. The scales can be found at www.traumameasuresglobal.com/depression and www.traumameasuresglobal.com/anxiety.

**Table 2a:** Derivation of the International Depression Questionnaire from ICD‐11 Description of Single Episode Depressive Disorder (6A70)

ICD‐11 Single Episode Depressive Disorder (6A70)	
Single Episode Depressive Disorder is characterized by the presence or history of one depressive episode when there is no history of prior depressive episodes. A depressive episode is characterized by a period of depressed mood or diminished interest in activities *(1) occurring most of the day*, *(2) nearly every day (3) during a period lasting at least two weeks* accompanied by *(4) other symptoms* such as difficulty concentrating, feelings of worthlessness or excessive or inappropriate guilt, hopelessness, recurrent thoughts of death or suicide, changes in appetite or sleep, psychomotor agitation or retardation, and reduced energy or fatigue.	1. Items 1 and 2 reference depressed mood or diminished interest in activities occurring “*for most of the day*?”
	2. Items are considered to be “endorsed” if response is “*Most days*” or “*Every day*.”
	3. Instructions state “*Over the last two weeks*, how frequently…”
	4. Items 3 to 9 measure each of these symptoms
*(5)* Depressive disorders must have “…*symptoms that significantly affect the individual's ability to function*.”	5. Functional impairment is assessed by “Have these experiences caused problems in personal, family, social, educational, occupational, or other important areas of your life?”

**Table 2b:** Self‐report measure of ICD‐11 Depressive Episode: The International Depression Questionnaire: **Over the last two weeks** , how frequently have you had the following feelings, thoughts, and behaviors? Please circle the appropriate number to indicate your response.

Never	Only a few days	Half the days	Most days	Every day
0	1	2	3	4

1. Felt down or depressed *for most of the day*?	0	1	2	3	4
2. Experienced less interest or pleasure from normal activities *for most of the day*?	0	1	2	3	4
3. Have had difficulty concentrating?	0	1	2	3	4
4. Had feelings of worthlessness or guilt?	0	1	2	3	4
5. Felt hopeless?	0	1	2	3	4
6. Had recurrent thoughts of death or suicide?	0	1	2	3	4
7. Have had changes in appetite or sleep?	0	1	2	3	4
8. Moved slower or felt more restless?	0	1	2	3	4
9. Experienced reduced energy or fatigue?	0	1	2	3	4
Have these experiences caused problems in personal, family, social, educational, occupational, or other important areas of your life? Yes ☐ No ☐					

For both questionnaires the instructions explicitly state the ICD‐11's time criterion, which is “a period lasting at least two weeks” for Depressive Episode (“*Over the last two weeks, how frequently have you had the following feelings, thoughts, and behaviors?*”) and “at least several months” for Generalized Anxiety Disorder (“*Over the last several months, how frequently have you had the following feelings, thoughts, and behaviors?*”) Tables 3a and 3b.

**Table 3a:** Derivation of the International Anxiety Questionnaire from ICD‐11 Description of Generalized Anxiety Disorder (6B00)

ICD‐11 Generalized Anxiety Disorder (6B00)	
Generalized Anxiety Disorder is characterized by marked symptoms of anxiety that *(1) persist for at least several months*, *(2) for more days than not*, manifested by (3) *either general apprehension (i.e.*, “*free‐floating anxiety*”*) or excessive worry* focused on multiple everyday events, most often concerning family, health, finances, and school or work, *(4) together with additional symptoms* such as muscular tension or motor restlessness, sympathetic autonomic over‐activity, subjective experience of nervousness, difficulty maintaining concentration, irritability, or sleep disturbance. *(5) The symptoms result in significant distress or significant impairment* in personal, family, social, educational, occupational, or other important areas of functioning.	1. Instructions sate “*Over the last several months, how frequently*…”
	2. Items are considered to be “endorsed” if response is “*Most days*” or “*Every day*.”
	3. Apprehension and excessive worry assessed by items 1 and 2.
	4. Items 3 to 8 measure each of these symptoms
	5. Functional impairment is assessed by “Have these experiences caused problems in personal, family, social, educational, occupational, or other important areas of your life?”

**Table 3b:** Self‐report measure of ICD‐11 Generalized Anxiety Disorder—The International Anxiety Questionnaire (IAQ): **Over the last several months** , how frequently have you had the following feelings, thoughts, and behaviors? Please circle the appropriate number to indicate your response.

Never	Only a few days	Half the days	Most days	Every day
0	1	2	3	4

1. Felt nervous or anxious?	0	1	2	3	4
2. Worried a lot about different things?	0	1	2	3	4
3. Felt physically tense or agitated?	0	1	2	3	4
4. Felt your heart racing, difficulty breathing, stomach discomfort, or dry mouth?	0	1	2	3	4
5. Felt “on edge”?	0	1	2	3	4
6. Had difficulty concentrating?	0	1	2	3	4
7. Been easily annoyed by different things?	0	1	2	3	4
8. Experienced sleep disturbances?	0	1	2	3	4
Have these experiences caused problems in personal, family, social, educational, occupational, or other important areas of your life? Yes ☐ No ☐					

The ICD‐11 requirements for the frequency of experiencing depressed mood or diminished interest in activities is “…occurring most of the day, nearly every day”. The IDQ reflects this by having the first two items suffixed with “…*for most of the day*” and using response options 3 (Most days) and 4 (Every day) as being indicative of endorsement. The ICD‐11 requirements for the frequency of experiencing symptoms of anxiety is “…*for at least several months, for more days than not*”. The IAQ reflects this by having the instructions stating, “*Over the last several months, how frequently have you*….” and using response options 3 (Most days) and 4 (Every day) as being indicative of endorsement.

It is proposed that the IDQ and the IAQ can be scored to capture symptom severity and also to identity probable diagnostic cases (i.e., those that meet ICD‐11 diagnostic requirements). The severity scoring method simply involves summing the scores of the nine IDQ items and the eight IAQ items, producing possible ranges of scores from 0 to 36 (IDQ) and 0 to 32 (IAQ), respectively. No cut‐off scores are proposed, as “caseness” is defined by applying the ICD‐11 diagnostic algorithm for each disorder (described below).

The ICD‐11 CDDR for Depressive Episode requires “*The concurrent presence of at least five of the … characteristic symptoms occurring most of the day, nearly every day during a period lasting at least 2 weeks. At least one symptom from the Affective cluster must be present*”. This equates to endorsing (i.e., scoring 3 or 4 on the Likert scale) questions 1 or 2 (or both) from the IDQ, and a total of 5 or more items being endorsed. If these conditions are met, *and* the functional impairment questions is answered “Yes,” then the diagnostic requirements for ICD‐11 Depressive Episode have been met.

The ICD‐11 CDDR for Generalized Anxiety Disorder requires the “*Essential (Required) Features*” of either “*General apprehensiveness that is not restricted to any particular environmental circumstance (i.e., ‘free‐floating anxiety’)*” or “*Excessive worry (apprehensive expectation) about negative events occurring in several different aspects of everyday life (e.g., work, finances, health, family)*”. This equates to endorsing (i.e., scoring 3 or 4 on the Likert scale) questions 1 or 2 on the IAQ. It also states that these essential features should be “…*accompanied by additional characteristic symptoms*”. For continuity with ICD‐10 (WHO, 1993), the IAQ requires a total of 4 or more items to be endorsed with at least one from the essential features. If these conditions are met, and the functional impairment question is answered “Yes,” then the diagnostic requirements for ICD‐11 Generalized Anxiety Disorder have been met.

DSM‐IV Major Depressive Disorder (MDD): The PHQ‐9 (Kroenke et al., 2001) measures the nine symptoms of MDD described in DSM‐IV (APA, 1994). Participants are asked to indicate how often they have been bothered by each symptom over the last 2 weeks on a 4‐point Likert scale ranging from 0 (*Not at all*) to 3 (*Nearly every day*). Possible scores range from 0 to 27 with higher scores reflecting higher symptomatology. The recommended and commonly used cut‐off score of ≥10 was used to identify possible “caseness.” This cut‐off score has been shown to have adequate sensitivity (0.85) and specificity (0.89) for detecting cases of MDD (Kroenke et al., 2001). The psychometric properties of the PHQ‐9 scores have been widely supported (Manea et al., 2012), and the internal reliability of the scale scores in this sample was *α* = 0.94.

DSM‐IV Generalized Anxiety Disorder (GAD): The GAD‐7 (Spitzer et al., 2006) measures seven symptoms of GAD described in DSM‐IV (APA, 1994). It asks participants to indicate how often they have been bothered by the various symptoms over the last 2 weeks on a 4‐point Likert scale that ranges from 0 (*Not at all*) to 3 (*Nearly every day*). Possible scores range from 0 to 21 where higher scores reflect greater symptomatology, and the recommended cut‐off score of ≥10 was used to identify possible “caseness.” This cut‐off score has been shown to have adequate sensitivity (0.89) and specificity (0.82) for detecting cases of GAD (Spitzer et al., 2006). The psychometric properties of the GAD‐7 scores have been widely supported (Hinz et al., 2017), and the internal reliability of the scale scores in this sample was *α* = 0.96.

Mental health treatment seeking: Participants were provided with the following information: “Mental health difficulties are very common. It will help us understand our survey results if you would tell us whether you currently or have in the past received treatment (medication or talking therapies) for these kind of difficulties”. Options were provided, of which the participants were required to choose one of: (1) “I have never received treatment for mental health problems,” (2) “I have received treatment for mental health problems in the past,” (3) “I am currently receiving treatment for mental health problems,” (4) “I am currently receiving treatment for mental health problems but it has been canceled temporarily due to the lockdown,” (5) “I am currently on a waiting list to receive treatment for a mental health problem,” and (6) “Prefer not to answer.” Options 3 and 4 were collapsed into one category, and “Prefer not to answer” responses were treated as missing data, resulting in a 4‐category variable: (1) No treatment ever, (2) Treatment in the past, (3) Treatment currently, and (4) Treatment waiting‐list.

### 2.3 Data analysis

First, the distributions of each of the IDQ and IAQ item scor were examined. The percentages of each response category were reported, along with the mean scores and percentage endorsements (score ≥ 3) as summary statistics. Item‐total correlations were also calculated and expected to exceed the minimum acceptable value of ≥ 0.30 (Lamping et al., 2002). The summed scores were also calculated, and differences in sex, age, and mental health treatment seeking were tested using *t*‐test and one‐way analysis of variance (ANOVA). The IDQ‐PHQ‐9 and IAQ‐GAD‐7 correlations were calculated using Pearson product‐moment correlations.

Second, both 1‐ and 2‐parameter IRT models were fitted to the data for the IDQ and IAQ separately. Binary item scores (score ≥ 3 on the Likert scale) were used to reflect the fact that the diagnostic algorithm uses item endorsement and not the full scale scores. For the 2‐parameter model, discrimination and difficulty parameters were estimated for all items. The discrimination parameter is the probit regression that relates the latent variable, theta (*θ*), to the binary indicator where higher values indicate increased discriminatory power. Desirable discrimination levels would be “high” (1.35–1.69) or “very high” (>1.70) (Baker, 1985). The difficulty parameter is estimated as thresholds. The 1‐parameter model was also tested where the item discrimination parameters were constrained to be equal. If the 1‐ and 2‐parameter models do not differ in fit, then the 1‐parameter model was considered the better model on the basis of parsimony. The DIFFTEST function (Asparouhov et al., 2006) from the software package Mplus 8.2 (Muthén & Muthén, 2018) was used to test for differences in model fit. These models were estimated using the robust weighted least squares estimator (WLSMV) with a nonlinear probit link based on the tetrachoric correlation matrix of latent continuous response variables. Model fit was assessed using common fit indices, and standard requirements were used to determine acceptable model fit: a nonsignificant chi‐square (*χ* ^*2*^), Comparative Fit Index (CFI) and Tucker‐Lewis Index (TLI) values ≥ .90, and Root Mean Square Error of Approximation (RMSEA) and Standardized Root Mean Residual (SRMR) values ≤ 0.08 and ≤ 0.05 indicating acceptable and excellent model fit, respectively.

Third, the prevalence estimates for ICD‐11 Depressive Episode and ICD‐11 Generalized Anxiety Disorder were calculated, and associations with sex, age, and mental health treatment were assessed using Pearson's *χ* ^*2*^ tests. Proportions of people exceeding the ≥10 cut‐off scores on the PHQ‐9 and the GAD‐7 were compared to the estimates obtained from the IDQ and the IAQ.

## 3 RESULTS

### 3.1 Descriptive statistics

The responses to the IDQ and IAQ items are reported in Table 4.

**Table 4:** Item response and summary statistics for the IDQ and IAQ

	Scale value					
	0	1	2	3	4	% Endorsed	Mean (SD)	Item‐total correlation
	Never	Only a few days	Half the days	Most days	Every day			
Depressive episode								
1. Felt down or depressed *for most of the day*?	50.2%	26.6%	10.1%	8.5%	4.6%	13.1%	0.91 (1.16)	0.86
2. Experienced less interest or pleasure from normal activities *for most of the day*?	49.6%	26.1%	10.0%	9.7%	4.6%	14.3%	0.94 (1.18)	0.88
3. Have had difficulty concentrating?	51.1%	24.7%	10.9%	8.7%	4.6%	13.3%	0.91 (1.17)	0.86
4. Had feelings of worthlessness or guilt?	60.3%	17.2%	8.8%	8.7%	5.0%	13.8%	0.81 (1.20	0.87
5. Felt hopeless?	60.0%	17.4%	9.1%	7.8%	5.6%	13.5%	0.82 (1.22)	0.88
6. Had recurrent thoughts of death or suicide?	74.9%	9.6%	6.8%	5.8%	3.0%	8.7%	0.52 (1.04)	0.77
7. Have had changes in appetite or sleep?	53.0%	23.4%	10.7%	8.5%	4.5%	12.9%	0.88 (1.17)	0.81
8. Moved slower or felt more restless?	61.3%	17.4%	9.7%	8.4%	3.2%	11.6%	0.75 (1.19)	0.83
9. Experienced reduced energy or fatigue?	42.6%	27.0%	11.8%	11.3%	7.3%	18.6%	1.14 (1.27)	0.80
Generalized anxiety disorder								
1. Felt nervous or anxious?	37.5%	35.9%	10.7%	9.8%	6.1%	15.8%	1.11 (1.19)	0.86
2. Worried a lot about different things?	37.3%	34.7%	10.5%	10.9%	6.6%	17.5%	1.15 (1.22)	0.87
3. Felt physically tense or agitated?	47.3%	27.6%	10.7%	9.8%	4.7%	14.4%	0.97 (1.18)	0.90
4. Felt your heart racing, difficulty breathing, stomach discomfort, or dry mouth?	61.1%	18.9%	9.2%	7.0%	3.7%	10.7%	0.73 (1.12)	0.82
5. Felt “on edge”?	49.6%	25.4%	10.8%	9.1%	5.1%	14.1%	0.95 (1.19)	0.89
6. Had difficulty concentrating?	49.4%	25.8%	10.3%	9.3%	5.2%	14.5%	0.95 (1.20)	0.86
7. Been easily annoyed by different things?	43.1%	30.8%	12.0%	9.3%	4.8%	14.1%	1.02 (1.16)	0.85
8. Experienced sleep disturbances?	39.3%	31.0%	11.6%	11.8%	6.4%	18.2%	1.15 (1.24)	0.75

Abbreviations: IAQ, International Anxiety Questionnaire; IDQ, International Depression Questionnaire.

The total scale scores for the IDQ covered the entire range of possible scores (0–36) with a mean of 7.66 (SD = 9.27). The distribution was positively skewed (*S* = 1.24, se = 0.05, *p* < 0.001). The total scale scores for the IAQ also covered the entire range of possible scores (0–32) with a mean of 8.02 (SD = 8.44), and the distribution was also positively skewed (*S* = 1.09, se = 0.05, *p* < 0.001).

The mean IDQ score was significantly higher for females (*M* = 8.03, SD = 9.08) than males (*M* = 7.19, SD = 9.40: *t* (2050) = 2.05, *p* = 0.041), and the effect size was small (*d*= 0.09). Similarly, the mean IAQ score was significantly higher for females (*M* = 8.75, SD = 8.34) than males (*M* = 7.18, SD = 8.44: *t* (2050) = 4.28, *p* < 0.001), and the effect size was small (*d* = 0.19). Age was negatively, weak‐to‐moderately, and significantly correlated with IDQ (*r* = −.33, *p* < 0.001) and IAQ (*r* = −.32, *p* < 0.001) scores. The correlations between the summed scores from the IDQ and the PHQ‐9 (*r* = .90, *p* < 0.001), and the IAQ and the GAD‐7 (*r* = .89, *p* < 0.001), were high, positive, and statistically significant.

One‐way ANOVAs indicted that there were significant differences across mental health treatment seeking status on IDQ scores (F [3, 1931] = 174.81, *p* < 0.001, *η*² = 0.21) and IAQ scores (F [3, 1931] = 201.16, *p* < 0.001, *η*² = 0.24). These effect sizes were “large”. The means of the IDQ and the IAQ across all levels of mental health treatment seeking status are reported in Table 5. Pairwise comparisons using the Scheffé post hoc tests indicated that all means were significantly different (*p* < 0.001) except for “Treatment currently” and “Treatment Waiting List” for both IDQ and IAQ.

**Table 5:** Mean scores on the IDQ and IAQ by treatment seeking status

	*N*	IDQ		IAQ	
		Mean	SD	Mean	SD
No treatment ever	1313	4.88	(7.41)	5.29	(6.77)
Treatment in the past	402	10.24	(9.52)	11.22	(8.39)
Treatment currently	170	17.61	(9.19)	17.22	(7.93)
Treatment Waiting List	50	17.98	(10.86)	17.10	(9.53)

*Note*: Missing data *n* = 123 (6.0%).

Abbreviations: IAQ, International Anxiety Questionnaire; IDQ, International Depression Questionnaire.

### 3.2 IRT and reliability results

The 1‐parameter IRT model for the IDQ provided adequate fit (χ^2^(35) = 247.68, *p* < 0.001; RMSEA = 0.054 (90% CI 0.048, 0.061); CFI = 0.994; TLI = 0.994; SRMR = 0.033) as did the 2‐parameter model (χ^2^ (27) = 198.53, *p* < 0.001; RMSEA = 0.056 (90% CI 0.048, 0.063); CFI = 0.995; TLI = 0.994; SRMR = 0.028). The DIFFTEST indicated that the models differed significantly Δ*χ* ^2^ = 65.61, Δ*df* = 8, *p* < 0.001), so the 2‐parameter model (with varying difficulty and discrimination parameters) was considered the better model.

The 1‐parameter IRT model for the IAQ provided adequate fit (*χ* ^2^ (27) = 247.68, *p* < 0.001; RMSEA = 0.064 (90% CI 0.057, 0.072); CFI = 0.995; TLI = 0.994; SRMR = 0.042) as did the 2‐parameter model (*χ* ^2^ (20) = 74.027, *p* < 0.001; RMSEA = 0.036 (90% CI 0.028, 0.045); CFI = 0.999; TLI = 0.998; SRMR = 0.016). The DIFFTEST indicated that the models differed significantly Δ*χ* ^2^ = 127.56, Δ*df* = 7, *p* < 0.001), so the 2‐parameter model was considered the better model. The estimates from these models are reported^2^ in Table 6.

**Table 6:** Item Response Theory Model Estimates for the IDQ and IAQ

	Item Parameters (se)				
	IDQ			IAQ	
	Discrimination	Difficulty		Discrimination	Difficulty
Item 1	2.864 (0.225)	1.190 (0.040)	Item 1	3.369 (0.295)	1.044 (0.036)
Item 2	3.127 (0.252)	1.121 (0.038)	Item 2	3.291 (0.279)	0.977 (0.035)
Item 3	2.408 (0.176)	1.204 (0.042)	Item 3	3.364 (0.295)	1.107 (0.037)
Item 4	2.911 (0.232)	1.154 (0.039)	Item 4	2.237 (0.177)	1.359 (0.047)
Item 5	3.159 (0.266)	1.159 (0.039)	Item 5	3.489 (0.307)	1.117 (0.037)
Item 6	2.035 (0.162)	1.511 (0.054)	Item 6	2.433 (0.180)	1.143 (0.040)
Item 7	1.972 (0.141)	1.267 (0.046)	Item 7	2.298 (0.168)	1.174 (0.042)
Item 8	2.256 (0.171)	1.307 (0.045)	Item 8	1.571 (0.102)	1.077 (0.045)
Item 9	2.204 (0.147)	0.980 (0.038)			

Abbreviations: IAQ, International Anxiety Questionnaire; IDQ, International Depression Questionnaire.

**Figure 1:**
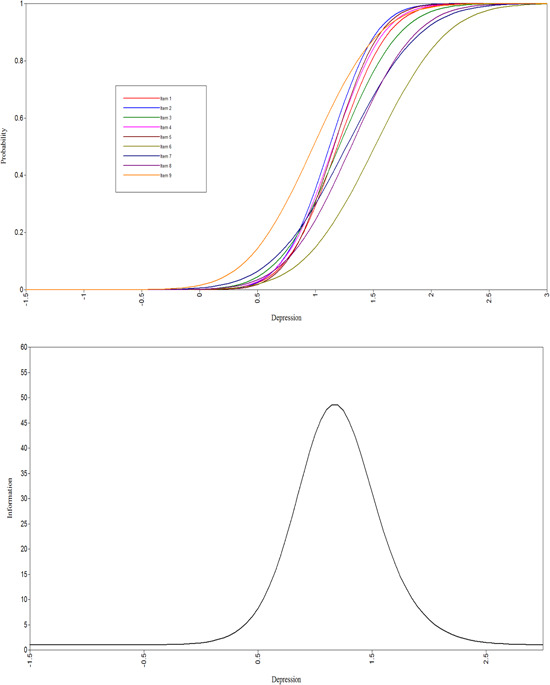
Item characteristic curves and total information curves for IDQ. IDQ, International Depression Questionnaire.

**Figure 2:**
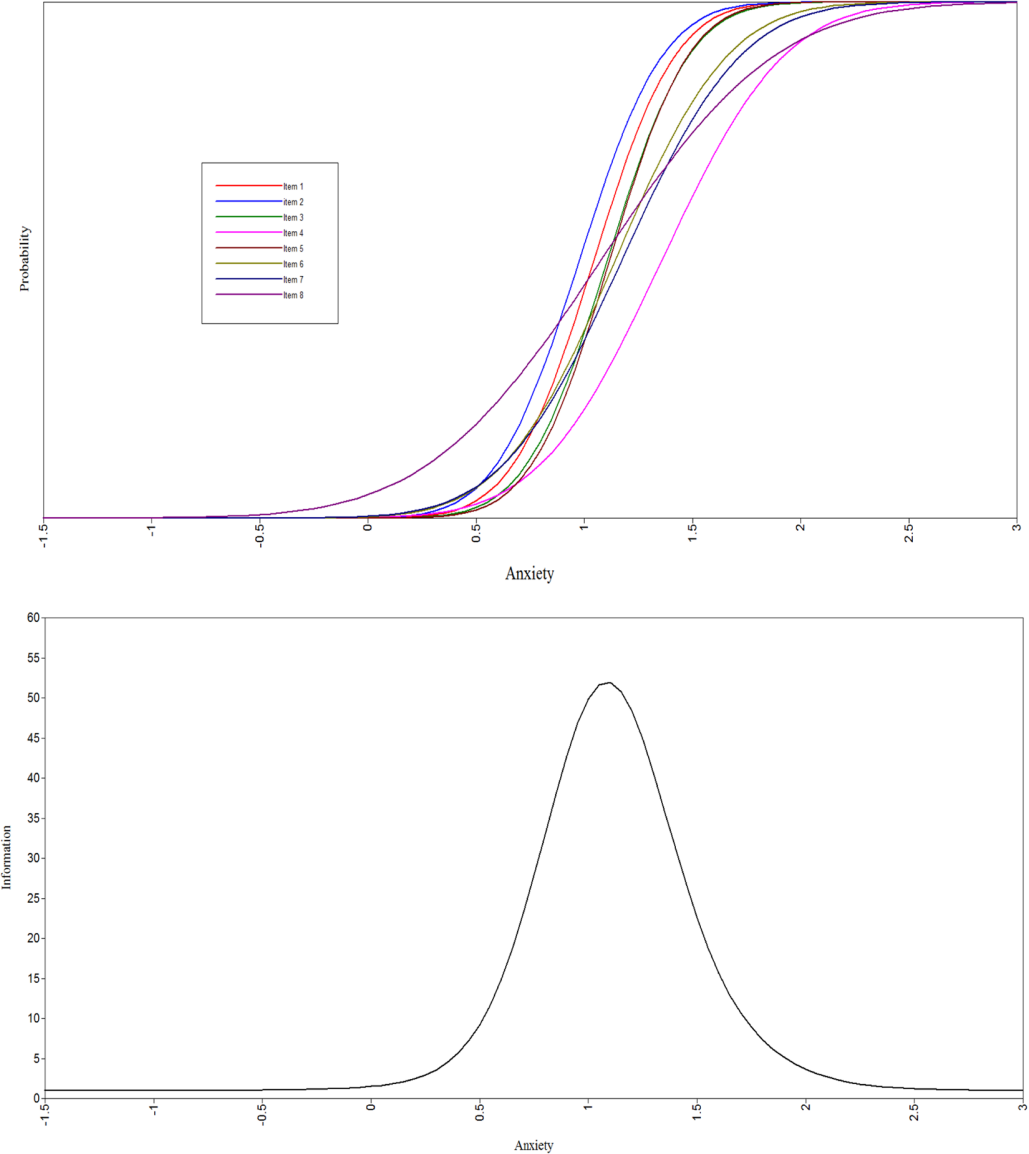
Item characteristic curves and total information curves for IAQ. IAQ, International Anxiety Questionnaire.

The IDQ discrimination parameter estimates were all statistically significant, and highest for item 5 (“Felt hopeless”) and item 2 (“Experienced less interest or pleasure from normal activities for most of the day”). The difficulty parameter estimates ranged from 0.980 for item 9 (“‘Experienced reduced energy or fatigue”) to 1.511 for item 6 (“Had recurrent thoughts of death or suicide”). Overall, the difficulty estimates indicate that these items are performing well at levels approximately 1 or more standard deviations above the mean of the underlying latent variable of depression. The item characteristic curves and total information curves are shown in Figures 1 and 2.

The IAQ discrimination parameter estimates were all statistically significant, and highest for item 5 (“Felt on edge”), item 3 (“Felt nervous or anxious for several months”), and item 1 (“Felt nervous or anxious for several months”). The difficulty parameter estimates ranged from 0.977 for item 2 (“Worried a lot about different things *for several months*?”) to 1.359 for item 4 (“Felt your heart racing, difficulty breathing, stomach discomfort, or dry mouth?”). Like the IDQ, the difficulty estimates indicate that these items are performing well at levels approximately 1 or more standard deviations above the mean of the underlying latent variable of anxiety.

The internal reliability of the IDQ (*ω* = 0.96) and the IAQ (*ω* = 0.96) scale scores were both high.

### 3.3 Prevalence estimates

In total, 7.4% (95% CI = 6.3%, 8.6%) of the sample met diagnostic requirements for ICD‐11 Depressive Episode, and 7.1% (95% CI = 5.9%, 8.2%) met requirements for ICD‐11 Generalized Anxiety Disorder. Overall, 9.8% (95% CI = 8.5%, 11.1%) met requirements for either disorder, 2.3% met requirements for Generalized Anxiety Disorder only, 2.7% for Depressive Episode only, and 4.8% for both. There was a significant association between meeting requirements for ICD‐11 depressive disorder and ICD‐11 generalized anxiety (*χ* ^2^ [1] = 813.58, *p* < 0.001). Of those who screened positive for ICD‐11 depressive disorder, 64.1% also screened positive for ICD‐11 generalized anxiety. Of those who screened positive for ICD‐11 generalized anxiety, 67.1% also screened positive for ICD‐11 depressive disorder.

There was no significant sex difference in ICD‐11 Depressive Episode (Males = 7.8%, Females = 6.9%: *χ* ^2^ [1] = 0.62, *p* = 0.430) or ICD‐11 Generalized Anxiety Disorder (Males = 7.6%, Female = 6.5%: χ^2^ [1] = 0.34, *p* = 0.340). Participants who screened positive for ICD‐11 depressive disorder were significantly younger (*M* = 36.15 years, SD = 10.99) than those who did not (*M* = 46.70 years, SD = 15.85: *t* [056] = 8.075, *p* < 0.001), and the effect size was medium‐to‐large (*d* = 0.68). Participants who screened positive for ICD‐11 generalized anxiety were also significantly younger (*M* = 37.64 years, SD = 11.41) than those who did not (*M* = 46.54 years, SD = 15.90: *t* [2056] = 6.619, *p* < 0.001), and the effect size was medium‐to‐large (*d* = 0.57).

There was a significant association between screening positive for ICD‐11 Depressive Episode and mental health treatment seeking (*χ* ^2^ [3] = 128.01, *p* < 0.001). Of those who met diagnostic requirements, a higher percentage were currently receiving treatment (21.8%) or on a waiting list (32.0%) compared to those who had treatment in the past (9.5%) or had never received treatment (3.7%). Similarly, there was a significant association between screening positive for ICD‐11 Generalized Anxiety Disorder and mental health treatment seeking (χ^2^ [3] = 190.80, *p* < 0.001). A higher percentage of those that met diagnostic requirements were currently receiving treatment (25.3%) or were on a waiting list (38.0%) compared to those who were treated in the past (8.5%) or had never received treatment (3.1%).

The percentage of the sample that exceeded the PHQ‐9 cut‐off for depression was 25.5% (95% CI = 23.6%, 27.4%), and the percentage that exceeded the GAD‐7 cut‐off for generalized anxiety was 20.7% (95% CI = 18.9%, 22.4%). There was a strong association between “caseness” based on the IDQ and the PHQ‐9 (*χ* ^2^ [1] =  478.73, *p* < 0.001) with 95.4% of the PHQ‐9 cases also being IDQ cases. Likewise, there was also a strong association between “caseness” based on the IAQ and the GAD‐7 (*χ* ^2^ [1] = 577.330, *p* < 0.001) with 86.7% of the GAD‐7 cases also being IAQ cases.

## 4 DISCUSSION

The goal of this study was to develop brief, easy‐to‐use, and freely available self‐report measures of ICD‐11 Depressive Episode and ICD‐11 Generalized Anxiety Disorder, and test their psychometric properties in a nationally representative general population sample. Our results provide preliminary support for the reliability and the validity of the IDQ and the IAQ scores, and indicate that these measures can be used to identify adults likely to be suffering from these disorders.

In a general adult population sample it would be expected that the scale items should be able to generate scores along the depression and anxiety continua, representing the absence of symptoms through to the highest levels of severity with all symptoms being endorsed. The distribution of scores on the IDQ and IAQ provided evidence to support this assumption as all response categories were used by the participants and, as expected, the scores were positively skewed. Furthermore, the scores were also homogeneous as indicated by the high levels of item‐total correlations. The homogeneous nature of the items was confirmed with the high estimates of internal reliability for the IDQ and IAQ scores. These are desirable characteristics of item‐level scores, and a necessary prerequisite for further psychometric analyses (Clark & Watson, 1995; Lamping et al., 2002).

Construct validity was supported by means of a 2‐parameter IRT model based on the scores from the IDQ and IAQ. The fit statistics supported the hypothesis of uni‐dimensionality, and the discrimination parameters indicated that the items on both scales performed well. For the IDQ, the two items with the highest discrimination were those measuring anhedonia (item 2), one of the two core affective symptoms required for diagnosis, and hopelessness (item 5). Previous research has identified hopelessness as an important factor in distinguishing depressed from nondepressed participants (McGlinchey et al., 2006). Indeed, one of the main differences between ICD‐11 and DSM‐5 is that in the latter hopelessness is a descriptors of depressed mood rather than separate symptom. With regard to the IAQ, the discrimination for items 1 (Felt nervous or anxious), 2 (Worried a lot about different things), 3 (Felt physically tense or agitated) and 5 (Felt ‘on edge’) were all high, indicating that the two core symptoms (items 1 & 2) necessary for diagnosis are operating well.

The rates of ICD‐11 Depressive Episode and ICD‐11 Generalized Anxiety Disorder derived from the IDQ and IAQ were 7.4% and 7.1%, respectively. These are slightly higher than the most recent UK population prevalence estimates from the Adult Psychiatric Morbidity Survey 2014 (McManus et al., 2016) which found that the prevalence of past‐week generalized anxiety disorder was 5.9% and past‐week depression was 3.3%. These figures were based on a structured clinical interview, the Clinical Interview Schedule (CIS‐R: Levis et al., 2020; Lewis et al., 1992) using the ICD‐10 diagnostic requirements. Structured clinical interviews tend to produce lower prevalence estimates than self‐reports (Thombs et al., 2018), and the past‐week timeframe is also likely to account for the slightly lower prevalence estimates. Taking these factors into account, the estimates based on the IDQ and IAQ appear to be reasonably similar to those from an ICD‐10 derived structured clinical interview. The slightly higher rates produced by the IDQ/IAQ relative to the CIS‐R may be partly attributable to the psychological consequences of COVID‐19 as there is evidence that rates of depression and anxiety increased somewhat after the pandemic (Patel et al., 2022), however, this effect has not been uniform across the entire population (Shevlin et al., 2022) and thus, such an interpretation should be made cautiously.

In contrast to the conservative estimated prevalence rates of ICD‐11 depressive disorder and generalized anxiety identified by the IDQ and IAQ, respectively, one‐quarter of the sample exceeded the PHQ‐9 threshold for depression (25.5%), and one‐fifth exceeded the GAD‐7 threshold for anxiety (20.7%). The largest study to date that has evaluated the diagnostic accuracy of the PHQ‐9 (Levis et al., 2019) concluded that the recommended and commonly used cut‐off score of 10 maximized combined sensitivity and specificity but also produced high levels of false positives (approximately 50% in a primary care setting). We propose that the close adherence to the ICD‐11 symptoms and application of the diagnostic algorithm, rather than a cut‐off score, would reduce the negative predicted value of the IDQ and the IAQ; although this is for future research to test. Encouragingly, the vast majority of individuals (>85%) who screened positive for depression and anxiety on the PHQ‐9 and GAD‐7 respectively, met diagnostic requirements for ICD‐11 Depressive Episode and ICD‐11 Generalized Anxiety Disorder on the IDQ and IAQ.

There was a significant association between meeting the requirements for ICD‐11 Depressive Episode and ICD‐11 Generalized Anxiety Disorder, and this was expected; the positive association between depression and anxiety at both the diagnostic and symptom level has been frequently documented (e.g., Jacobson & Newman, 2017; Möller et al., 2016). Indeed, this overlap has been widely acknowledged clinically (Kalin, 2020) and studies based on large general population samples have found that co‐occurring clinically relevant anxiety and depression was more common than either anxiety or depression alone (e.g., Goldberg et al., 2017; Shevlin et al., 2022). Indeed, this is what was found in this study as more people met requirements for ICD‐11 depressive disorder and generalized anxiety (4.6%) than depressive disorder alone (2.7%) and generalized anxiety alone (2.3%). There is a provision in the ICD‐11 to be able to identify “Prominent anxiety symptoms in Mood Episodes” (6A80.0) and “Mixed depressive and anxiety disorder” (6A73) which can accommodate the co‐occurrence of symptoms from both disorders. The IDQ and IAQ provide the opportunity to assess these symptoms and disorders.

There are some limitations of this study. First, as this study provides only initial evidence of validity of these newly developed measures, future research is needed to establish the degree of agreement between clinical interview assessment and IDQ/IAQ scores. Second, while the study did not used data from a probability‐based sample, the sample characteristics of age, sex, and household income were representative of the UK adult population. Third, future research on the performance of these scales is required in clinical setting with participants displaying clinically significant levels of mood and anxiety distress. Given the international focus of the ICD‐11, cross‐cultural analyses are also required.

In conclusion, the IDQ and the IAQ are brief self‐report measures directly derived from the ICD‐11 diagnostic descriptions of Depressive Episode and Generalized Anxiety Disorder and freely available to all interested parties. Initial findings from analyses based on data from a large nationally representative sample of the UK adult population are encouraging: They indicate that these scales (1) produced adequate variability in scale scores, (2) have high levels of internal consistency, (3) have high/very high levels of discrimination, (4) tap information at the upper end of the underlying distributions, and (5) appear to be related to mental health help‐seeking.

## CONFLICT OF INTEREST

The authors declare no conflict of interest.

### TRANSPARENT PEER REVIEW

The peer review history for this article is available at https://publons.com/publon/10.1002/jclp.23446

## Data Availability

Data are publicly available at https://osf.io/qv47z/

## ENDNOTES

1 The IDQ/IAQ and the PHQ‐9/GAD‐7 were not in consecutive parts of the survey and the order in which they were presented was randomized to remove any order effects.

2 The models were also estimated using the data from the original 5‐category responses. For the IDQ the 1‐parameter (*χ* ^2^ (35) = 1347.37, *p* < 0.001; RMSEA = 0.135 (90% CI 0.129, 0.411); CFI = 0.989; TLI = 0.989; SRMR = 0.030) and 2‐parameter (*χ* ^2^ (27) = 990.40, *p* < 0.001; RMSEA = 0.132 (90% CI 0.125, 0.139); CFI = 0.992; TLI = 0.989; SRMR = 0.019) models both had RMSEA values that were too high. A similar pattern of fit statistics was evident for the IAQ: 1‐parameter (*χ* ^2^ (27) = 1279.52, *p* < 0.001; RMSEA = 0.150 (90% CI 0.143, 0.157); CFI = 0.990; TLI = 0.989; SRMR = 0.034) and 2‐parameter (*χ* ^2^ (20) = 618.21, *p* < 0.001; RMSEA = 0.121 (90% CI 0.112, 0.129); CFI = 0.995; TLI = 0.983; SRMR = 0.014).
